# Compare Analysis of Codon Usage Bias of Nuclear Genome in Eight Sapindaceae Species

**DOI:** 10.3390/ijms26010039

**Published:** 2024-12-24

**Authors:** Yuxuan Song, Meng Shen, Fuliang Cao, Xiaoming Yang

**Affiliations:** Co-Innovation Center for Sustainable Forestry in Southern China, Nanjing Forestry University, Nanjing 210037, China

**Keywords:** Sapindaceae, codon usage bias, nature selection, mutation, evolution

## Abstract

Codon usage bias (CUB) refers to the different frequencies with which various codons are utilized within a genome. Examining CUB is essential for understanding genome structure, function, and evolution. However, little was known about codon usage patterns and the factors influencing the nuclear genomes of eight ecologically significant Sapindaceae species widely utilized for food and medicine. In this study, an analysis of nucleotide composition revealed a higher A/T content and showed a preference for A/T at the third codon position in the eight species of Sapindaceae. A correspondence analysis of relative synonymous codon usage explained only part of the variation, suggesting that not only natural selection but also various other factors contribute to selective constraints on codon bias in the nuclear genomes of the eight Sapindaceae species. Additionally, ENC-GC3 plot, PR2-Bias, and neutrality plot analyses indicated that natural selection exerted a greater influence than mutation pressure across these eight species. Among the eight Sapindaceae species, 16 to 26 optimal codons were identified, with two common high-frequency codons: AGA (encoding Arg) and GCU (encoding Ala). The clustering heat map, which included the 8 Sapindaceae species and 13 other species, revealed two distinct clusters corresponding to monocots and dicots. This finding suggested that CUB analysis was particularly effective in elucidating evolutionary relationships at the family level. Collectively, our results emphasized the distinct codon usage characteristics and unique evolutionary traits of the eight Sapindaceae species.

## 1. Introduction

The genetic code comprises the molecular mechanism used by cells to translate nucleotide sequences into proteins. Of the 64 codons in the standard genetic code, 61 encode the 20 standard amino acids, while the remaining 3 serve as stop codons [1]. The redundancy of the genetic code permits the same amino acid to be encoded by different synonymous codons. Specifically, tryptophan and methionine are encoded by a single codon each, while the remaining 18 amino acids are encoded by multiple synonymous codons, each specifying the same amino acid. Codon usage bias (CUB) refers to the preferential use of certain codons over others and is widespread across species [2]. CUB reflects the non-uniform use of codons in gene encoding and plays a role in gene regulation [3].

Several hypotheses have been proposed to explain the occurrence of CUB. Codon usage in a gene is shaped by the interplay of mutational bias, genetic drift, and natural selection [1,3,4]. The neutral theory suggests that mutational pressure at degenerate codon positions is neutral, leading to the nonuniform usage of synonymous codons for a specific amino acid [5]. Mutations in coding regions, particularly in the second or third nucleotide of a codon, that replace one synonymous codon with another, do not change the amino acid or the peptide’s sequence [6]. These synonymous mutations have no functional impact but lead to codon usage biases in genomes through selection during evolution [7]. Generally, gene expression levels are associated with CUB, and highly expressed proteins are mainly encoded by genes with optimal codons. Natural selection in highly expressed genes significantly influences codon usage across organisms [8]. Other key factors influencing innate CUB include base composition [9], base skewness [9], gene length [10], gene stability during replication [11], translational selection [12], protein secondary structure [13], and so on.

The Sapindaceae family encompasses 150 genera and approximately 2000 species, exhibiting a broad spectrum of plant forms ranging from herbs to vines, shrubs, and trees [14,15]. This family is predominantly distributed across tropical and subtropical regions. The economic significance of numerous Sapindaceae species is noteworthy, particularly for their contributions to food production, agriculture, pharmacology, and the natural products industry [15,16]. For example, fruits like litchi (*Litchi chinensis*), longan (*Dimocarpus longan*), and rambutan (*Nephelium lappaceum*) are prized for their taste and nutritional value. Several species are also valued for their secondary metabolites, such as saponins from soapberry (*Sapindus mukorossi*) and seed oil from yellowhorn (*Xanthoceras sorbifolium*), which have diverse industrial and pharmaceutical uses. Moreover, some species, like *Acer yangbiense*, are sought after for their durable and aesthetically pleasing timber, widely used in construction and furniture making. Other species, such as balloon vine (*Cardiospermum halicacahum*) and horse chestnut (*Aesculus chinensis*), are known for their medicinal properties, playing a role in traditional medicine across various cultures. With the development of next-generation sequencing technologies, several full genomes of the Sapindaceae plants mentioned above have been released [16], which will accelerate breeding programs, as well as ecological and evolutionary research.

As we enter the post-genomic era, characterized by a surge in genetic sequence data, it is crucial to analyze the generated databases to extract meaningful insights. Although advances in genome sequencing technology and increased research on plant genome codons have occurred, most studies on plant codon preference have focused on a single species, particularly chloroplast or mitochondrial genomes. Moreover, limited research has focused on CUB at the family level in plant species. This study presents a detailed examination of codon usage patterns and their determinants, utilizing the nuclear genomes of eight Sapindaceae species: *A. yangbiense*, *A. chinensis*, *C. halicacahum*, *S. mukorossi*, *X. sorbifolium*, *L. chinensis*, *N. lappaceum,* and *D. longan*. This understanding of codon usage patterns in Sapindaceae species will therefore aid in deciphering the evolutionary mechanisms of species’ genomes.

## 2. Results

### 2.1. Assessing the Correlation Between Codon Usage Metrics

**Figure 1:**
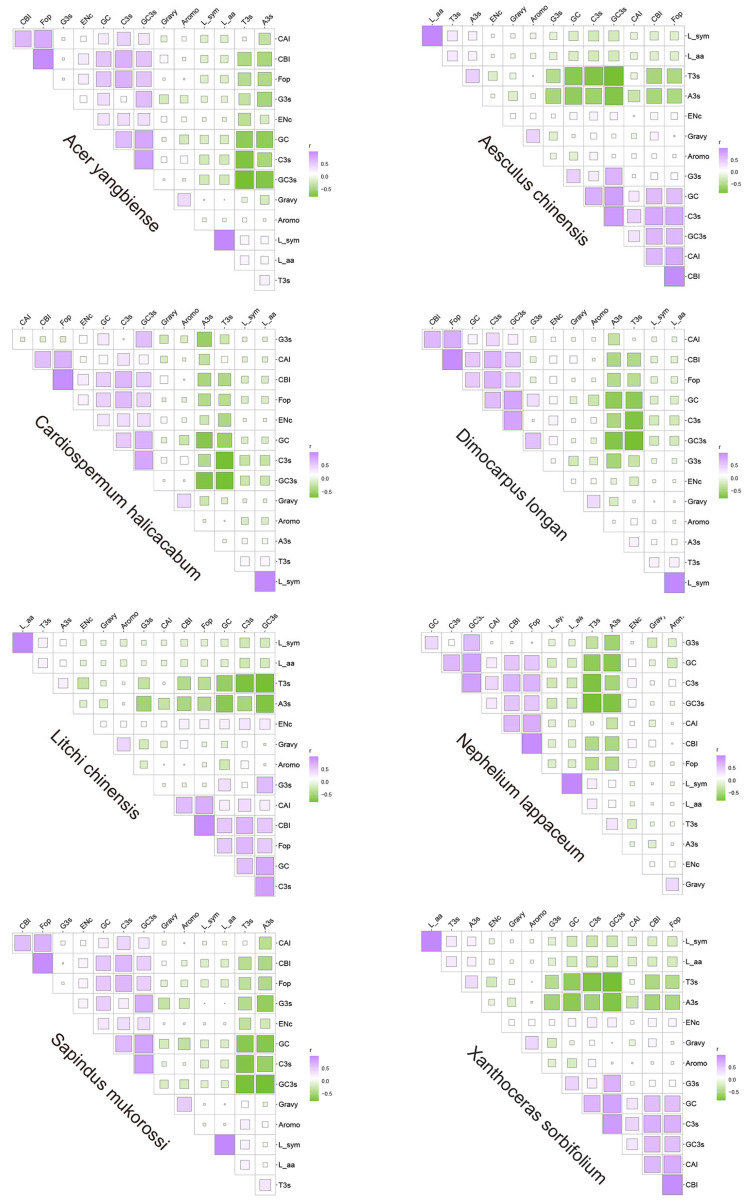
Correlation analysis is conducted among different indices across eight Sapindaceae species. Correlations are represented by the intensity of the colors, ranging from low to high. Purple indicates a positive correlation, while green indicates a negative correlation. Higher values represent stronger correlations. A3s, T3s, C3s, GC3s: composition of third synonymous codons. CAI: codon adaptation index. CBI: codon bias index. Fop: frequency of optimal codons. Nc: effective number of codons. L_sym: number of synonymous codons. L_aa: length of amino acids. Gravy: grand average of hydropathicity. Aromo: aromaticity.

A significant positive correlation was observed between GC content and GC3s in the eight Sapindaceae species (*p* < 0.01). Additionally, significant positive correlations were identified between GC, GC3s, and CBI (codon bias index) in these species. GC content showed a strong negative correlation with A3s/T3s and a significant positive correlation with C3s/G3s (*p* < 0.01). FOP (frequency of optimal codons) showed a significant positive correlation with CBI across all eight Sapindaceae species (*p* < 0.01). Moreover, a similar correlation was found between L_sym (number of synonymous codons) and L_aa (length of amino acids) in these species. These results indicated that nucleotide composition could influence the CUB of genes in Sapindaceae species (Figure 1 and Table S1).

### 2.2. Analysis of Nucleotide Composition and Codon Usage in Eight Sapindaceae Species

The GC content in CDSs across the eight Sapindaceae species ranged from 43.09% to 45.53%, with *C. halicacahum* having the lowest and *A. chinensis* the highest. In these species, CDSs exhibited a lower abundance of G and C nucleotides compared to A and T. Additionally, the average GC1 content surpassed that of GC3 and GC2 in all species, following the trend GC1 > GC3 > GC2 (Table 1). GC1 content ranged from 49.44% to 50.57%, with *A. yangbiense* showing the lowest and *A. chinensis* the highest. GC2 content ranged from 39.64% to 41.06%, with *A. yangbiense* showing the lowest and *S. mukorossi* the highest. GC3 content ranged from 39.59% to 45.17%, with *C. halicacahum* showing the lowest and *A. chinensis* the highest. Similar trends were observed at the third position of synonymous codons, with GC3s content ranging from 37.25% (*C. halicacahum*) to 43.05% (*A. chinensis*).

### 2.3. ENC-Plot Analysis

Codon bias in genes was typically assessed using ENC values. The average ENC values across the eight Sapindaceae species ranged from 51.20 ± 4.18 to 53.37 ± 4.11, with *C. halicacabum* showing the lowest and *A. chinensis* the highest. For the remaining species, the average ENC values were 51.99 ± 4.46 for *S. mukorossi*, 52.19 ± 3.95 for *L. chinensis*, 52.20 ± 3.95 for *D. longan*, 52.35 ± 4.08 for *A. yangbiense*, 52.53 ± 4.03 for *N. lappaceum*, and 52.75 ± 3.97 for *X. sorbifolium*, indicating a generally random codon usage pattern across the eight Sapindaceae species.

**Figure 2:**
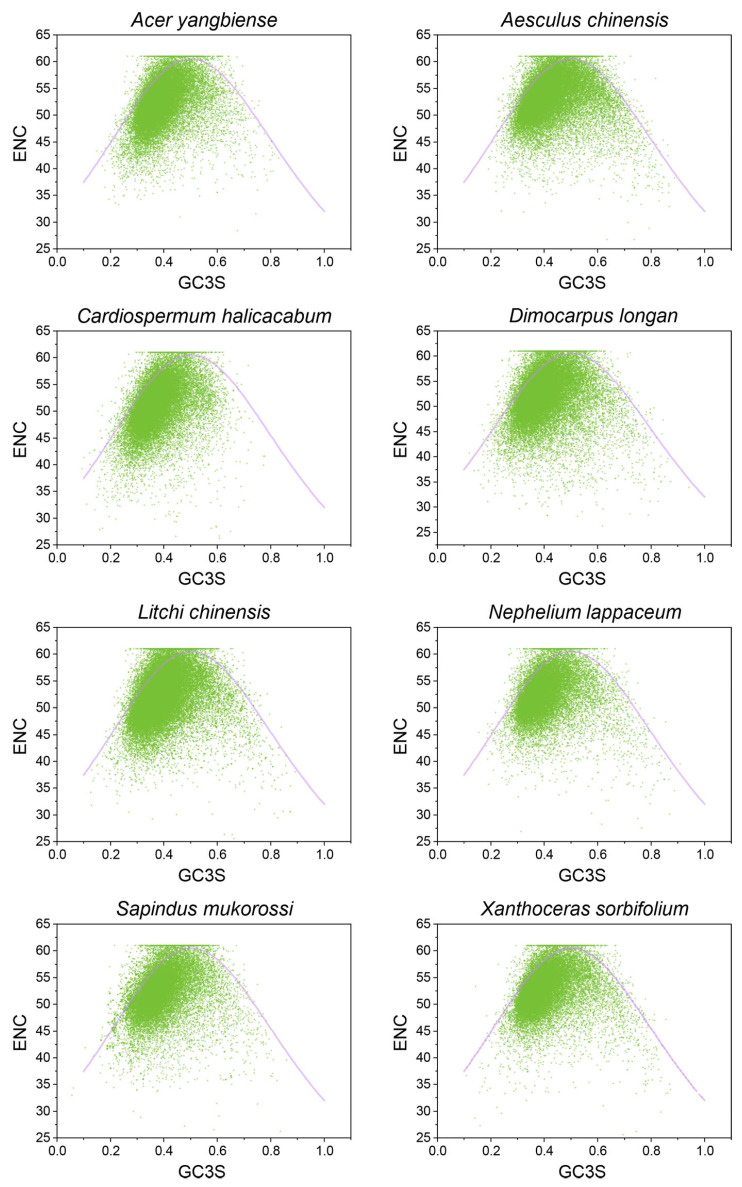
The ENC plot analysis is conducted on CDSs across the eight Sapindaceae species. The purple solid line in the plot represents the expected curve of positions of genes when the codon usage is only determined by the GC3s composition.

To explore the relationship between nucleotide composition and codon bias in Sapindaceae genomes, we performed an ENC-GC3 analysis. The results indicated that most CDSs in each species fell below the expected ENC plot curve, with only a small number aligning with it (Figure 2). This analysis suggested that selection pressure was the main factor influencing CUB in the eight Sapindaceae species, while mutational pressure appeared to affect only a limited number of CDSs.

To further estimate the differences between the observed and expected ENC values and confirm the influence of GC3s in Sapindaceae species, we calculated the (ENCexp − ENCobs)/ENCexp ratio. The analysis revealed that most (ENCexp − ENCobs)/ENCexp values ranged from −0.1 to 0.3, with over 55% falling between 0 and 0.1. These findings indicated that natural selection was the primary factor influencing codon usage in most CDSs of the eight Sapindaceae species, with some CDSs also exhibiting effects from mutational bias. This further suggested that codon bias formation in these genomes largely accounted for variations in GC3s (Table 2).

### 2.4. PR2 Plot Analysis

**Figure 3:**
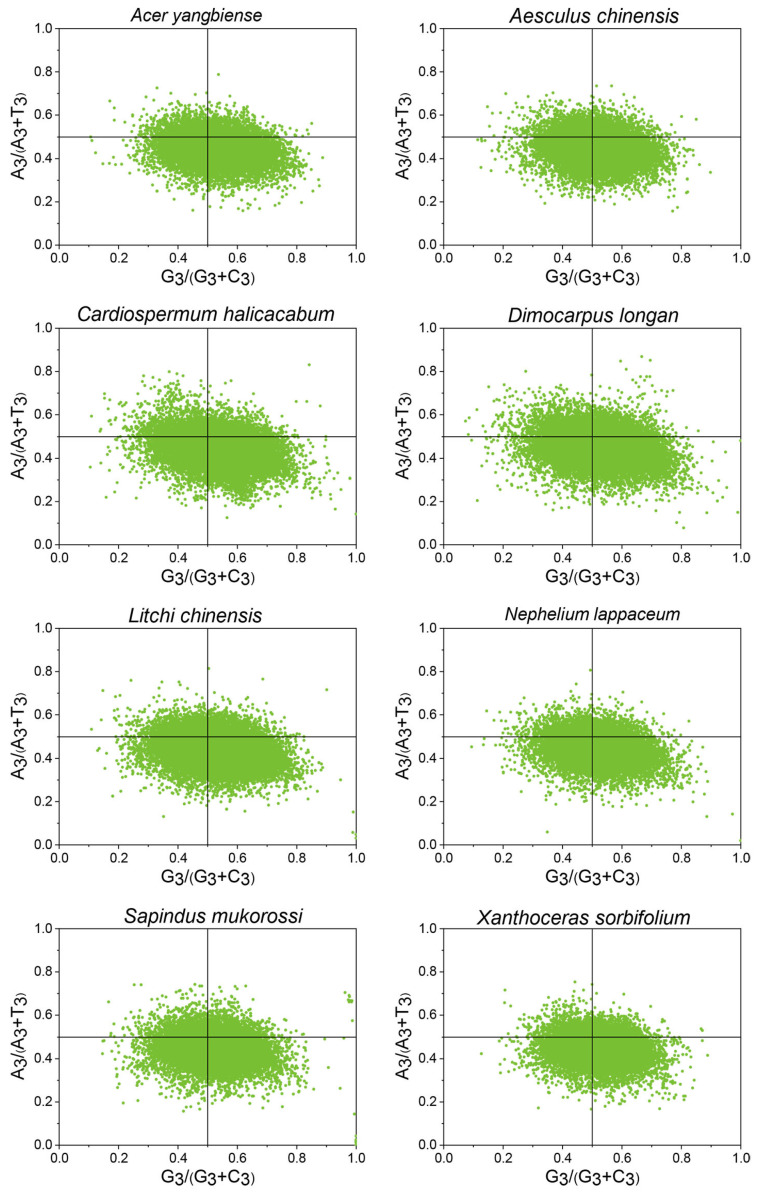
The PR2 plot analysis is performed on CDSs across eight Sapindaceae species. The purple solid line represents the regression line.

CUB is typically linked to the third base of the codon. We analyzed the frequencies of A/T and G/C at this position to assess the impact of mutational and selection pressures on codon usage in the genomes of eight Sapindaceae species. A discrepancy between A and T frequencies compared to C and G at the third codon position indicated that natural selection predominantly drove codon usage bias. In contrast, equal usage of A/T and G/C suggested the influence of mutational pressure. As illustrated in Figure 3, most genes in these species fell within the lower right quadrant (G3/(G3 + C3) < 0.5, A3/(A3 + T3) > 0.5), signifying that T occurred more frequently than A, while G was utilized more than C at the third codon position. This imbalance in A/T and G/C frequencies indicated that both mutation and natural selection contributed to codon usage bias in these eight Sapindaceae genomes.

### 2.5. Neutrality Plot Analysis

**Figure 4:**
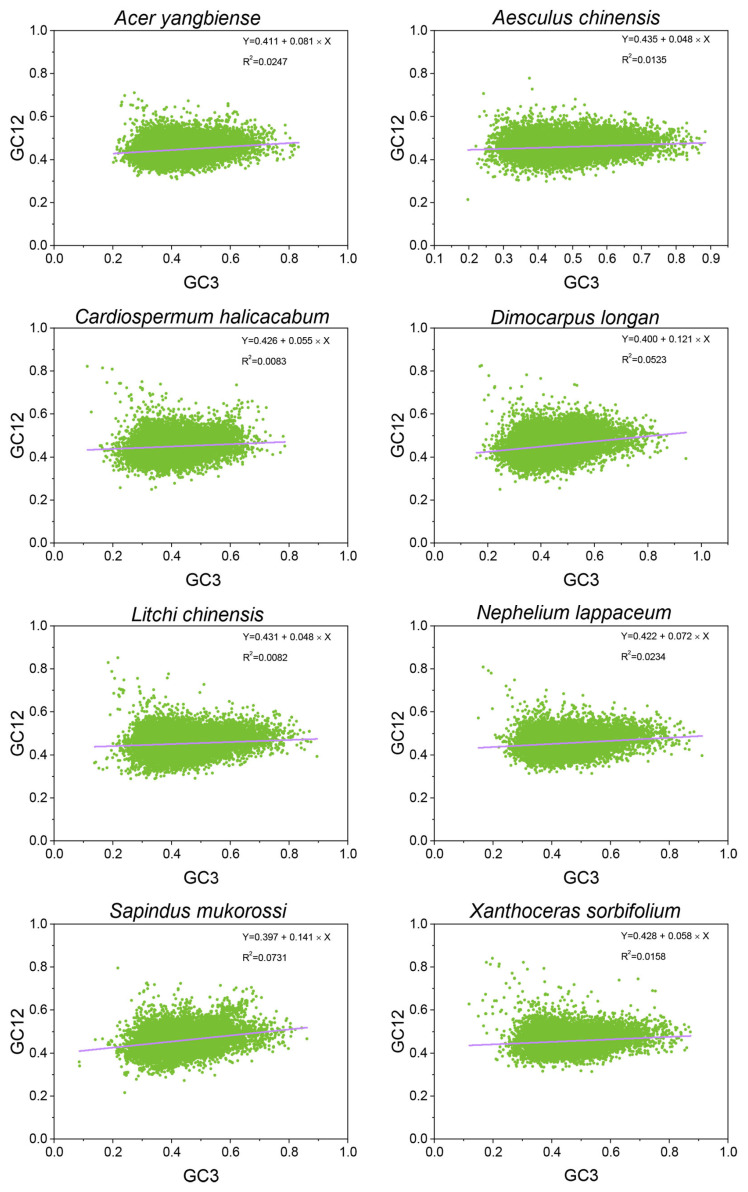
The neutrality plot analysis is conducted on the GC12 and GC3 of CDSs in eight Sapindaceae species. The purple line represents the regression line.

To identify the key factors influencing CUB in Sapindaceae genomes, we conducted a neutrality plot analysis of various species’ CDSs (Figure 4). The absolute value of the regression line’s slope typically indicated the extent of neutral mutation pressure on codon usage patterns. A slope near 1, with CDSs lately aligned along the diagonal, suggested that CUB was primarily driven by mutational pressure. As the slope approached 0, natural selection’s influence on CUB increased progressively. A neutrality plot analysis revealed there were correlations between GC12 and GC3 in all eight Sapindaceae species (*p* < 0.01), with slope values ranging from 0.048 (*A. chinensis*) to 0.141 (*S. mukorossi*)*,* and R^2^ values ranging from 0.0082 (*L. chinensis*) to 0.0731 (*S. mukorossi*). These results indicated that codon usage bias was minimally influenced by mutational pressure, while natural selection and other factors played a more significant role in these eight Sapindaceae species, consistent with the ENC-GC3s and PR2 plot analyses.

### 2.6. Correspondence Analysis

**Figure 5:**
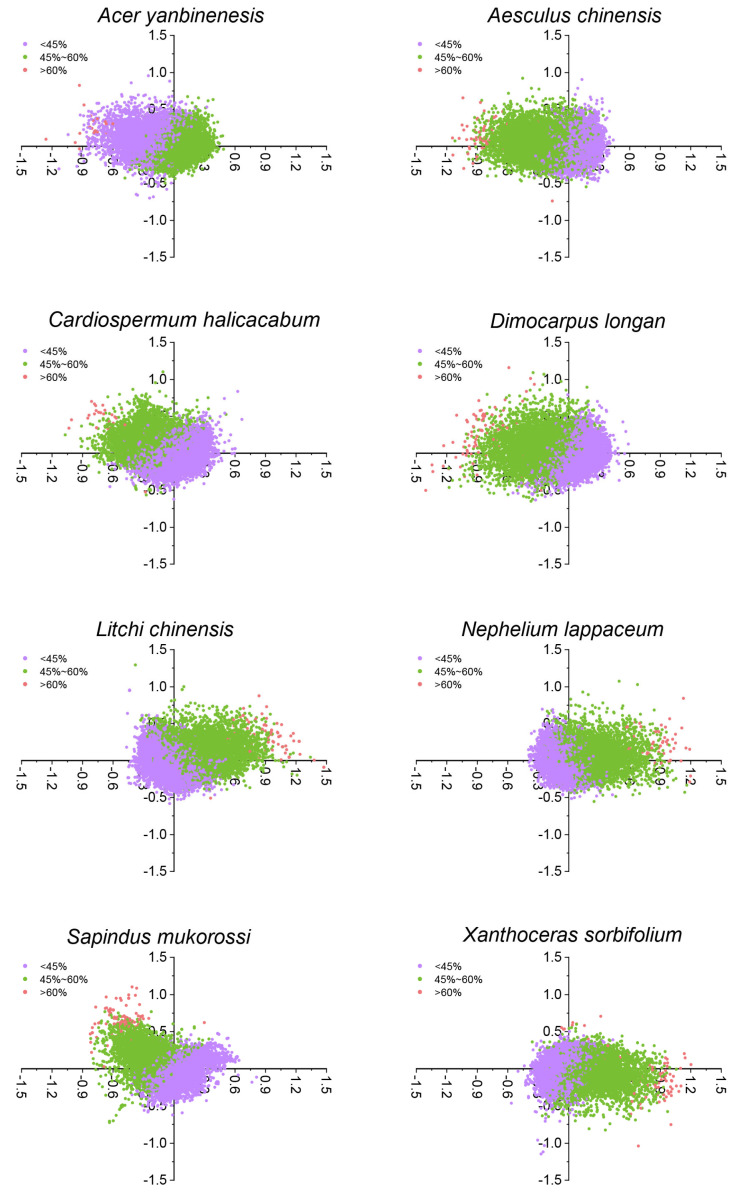
Correspondence analysis using the RSCU values of CDSs in eight Sapindaceae species. Purple, green, and red denote CDSs with GC content below 45%, between 45% and 60%, and above 60%, respectively.

COA is a multivariate statistical method used to analyze relationships between variables within a sample based on RSCU values. It was used to identify key factors influencing codon usage patterns in the genomes of eight Sapindaceae species. The first four axes accounted for 29.23% of the cumulative variation. Axis 1 was the main factor influencing CUB, accounting for 13.20% of the variation, with each subsequent axis explaining progressively less (Figure 5). To examine the impact of GC content on CUB, genes were color-coded by GC content in these species. The results showed that CDSs with a GC content below 45% or between 45% and 60% were mainly concentrated on both sides of the coordinate axis, while CDSs with a GC content exceeding 60% were primarily distributed on the right or left side of the axis (Figure 5). These results indicated that both selection pressure and gene mutation influenced CUB in Sapindaceae species’ genomes. Although eight Sapindaceae species shared similar overall codon usage patterns, CDSs in different species exhibited unique evolutionary traits in codon usage.

### 2.7. Optional Codon Analysis of Sapindaceae Species

**Figure 6:**
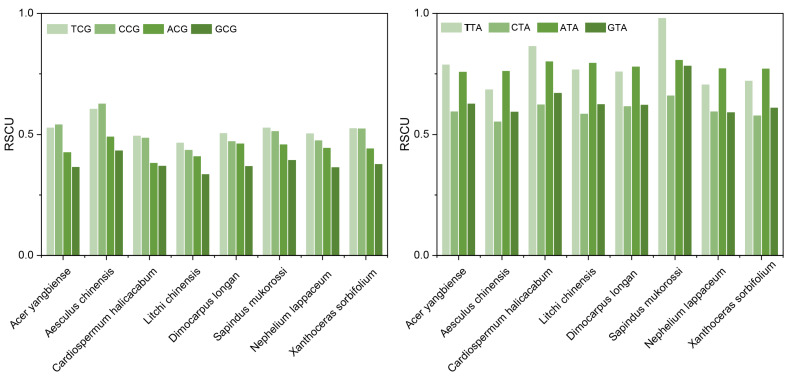
The RSCU value of NCG and NTA in eight Sapindaceae species.

Species such as *A. yangbiense*, *A. chinensis*, *C. halicacabum*, *D. longan*, *L. chinensis*, *N. lappaceum*, *S. mukorossi*, and *X. sorbifolium* exhibited 25, 16, 26, 19, 23, 18, 25, and 20 optimal codons (ΔRSCU > 0.08 and RSCU > 1), respectively (Table S2). Most of the optimal codons in these species ended with either A or T. However, four NTA and four NCG codons in the eight Sapindaceae species exhibited low RSCU values (Figure 6). Additionally, all Sapindaceae species had two common high-frequency codons, AGA and GCU, encoding arginine (Arg) and alanine (Ala), respectively. AGA was the most frequent codon among the eight Sapindaceae species (Table 3). For stop codons, the average RSCU values for TGA, TAA, and TAG were 1.264, 1.002, and 0.733, respectively, which indicated that the eight Sapindaceae species showed a preference for TGA as the stop codon.

### 2.8. Codon Usage Patterns of WRKY Gene Family Members in Eight Sapindaceae Species

Different WRKY gene family members were successfully identified across the eight Sapindaceae species (Table S3) and analyzed for their codon usage patterns. The results revealed that candidate sequences in the WRKY gene family exhibited distinct codon preferences, particularly at the third codon position. The overall GC content of these WRKY family members ranged from 42.27% to 45.52%, with *C. halicacabum* showing the lowest and *A. chinensis* the highest values. Among the codon positions, the average GC1 content was higher than GC3 and GC2 across all species. Specifically, GC1 content ranged from 45.93% to 48.72%. GC2 content ranged from 42.76% to 44.32%, while GC3 content ranged from 37.73% to 44.12%. These findings indicated a preference for A/T usage at the third codon position. Based on the RSCU analysis, we identified 28, 30, 30, 29, 29, 29, 30, and 29 high-frequency codons in *A. yangbiense*, *A. chinensis*, *C. halicacabum*, *D. longan*, *L. chinensis*, *N. lappaceum*, *S. mukorossi*, and *X. sorbifolium*, respectively (Table S4). Notably, the majority of these high-frequency codons terminate in A/T, indicating a preference for codons ending in A/T, which may be associated with enhanced translational efficiency. In summary, the WRKY gene family across the eight Sapindaceae species exhibited a pronounced preference for codons ending in A/T at the third position, mirroring the general codon usage patterns observed in the CDSs of these species. These findings underscore the importance of understanding codon preferences for optimizing gene expression and selecting appropriate expression systems.

### 2.9. Comparative Analysis of CUB Between Sapindaceae Species and Other Species

To better understand the relationship between GC3 variations in the eight Sapindaceae species, we calculated their Euclidean distances, which ranged from 0.102 to 0.597 (Figure S1). *A. chinensis* and *C. halicacabum* showed the largest Euclidean distance, indicating that they were the most distantly related species. In contrast, *N. lappaceum* and *X. sorbifolium* had the smallest Euclidean distance, suggesting a closer relationship. *A. yangbiense* exhibited a relatively larger distance from *A. chinensis* but was more closely related to *L. chinensis, D. longan,* and *S. mukorossi* due to their smaller Euclidean distances. These findings suggested that the similar GC3 patterns among most Sapindaceae species would be due to consistent mutation pressures.

**Figure 7:**
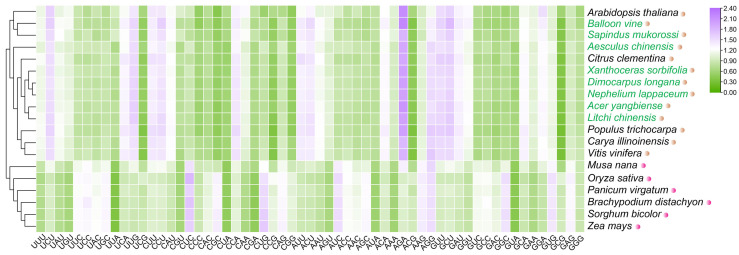
The heat map of RSCU, based on 59 codons from 21 species, is generated using Euclidean distance. The pink and orange color circles represent monocots and dicots, respectively. The eight Sapindaceae species are marked with a green font. Correlations are represented by the intensity of the colors, ranging from low to high. Green indicates a low correlation, while purple represents a positive correlation.

To explore changes in CUB among the eight Sapindaceae species during evolution, we conducted a comparative analysis that included 13 monocot and dicot species. We generated a clustering heat map to compare the RSCU values of 59 synonymous codons, excluding Met, Trp, and three stop codons, emphasizing evolutionary shifts in CUB across these species. The heat map (Figure 7) showed that angiosperms clustered into two main groups: monocots and dicots. Sapindaceae species were further divided into three clades. *S. mukorossi* and *B. vine* were closely related to *A. thaliana*, whereas the remaining six Sapindaceae species clustered together and showed a closer relationship, with mainly dicots, including *Citrus Clementina*, *Populus trichocarpa*, *Carya illinoinensis,* and *Vitis vinifera*. We further analyzed the GC distribution from 21 plant genomes to show that species within the same group exhibited a similar GC and GC3 content (Figure S2). The distribution showed significant variation across species and evolved over time, as confirmed by the results. In dicots, the GC3 content was around 0.4, while in monocots, it exceeded 0.5. It was hypothesized that GC-rich CDSs offered a selective advantage due to their potential for more complex gene regulation.

## 3. Discussion

CUB, a key factor in gene regulation and molecular evolution, is present across all organisms, including prokaryotes and eukaryotes [1,2]. However, the degree of preference for synonymous codons varies between species. Previous studies on CUB analysis mostly focused on a single species or gene family, primarily targeting chloroplast or nuclear genomes [9,17]. However, such studies rarely extended to a broader range of species, especially at the family and order levels, where genetic differences would be larger. This study aimed to analyze the nuclear genomes of eight Sapindaceae species to examine codon usage patterns and the evolutionary forces affecting codon usage bias, broadening its application in genome biology research.

Nucleotide composition, particularly GC content, is one of the key factors influencing CUB and reflects the overall trend of codon mutations, with the third codon position being the most indicative of genomic base composition [5]. Genomic GC content exhibits considerable variation among species due to differing mutational pressures. GC3 is commonly used to assess codon preference, as the third position of synonymous codons is influenced by selective pressure [18]. A notable difference in GC3 content exists between monocots and dicots. Monocots generally exhibit GC3 values exceeding 50% and preferentially use C/G codons, while dicots tend to favor A- and T-ending codons [9]. In Sapindaceae species, codon usage was biased toward A- and T-ending codons, with an average GC3 content ranging from 43.09% to 45.53% in their genomes. Therefore, the AT-biased genomic architecture in CDSs is associated with evolutionary fitness [19]. Our study observed a trend of increasing GC content from GC1 to GC3, although all three positions had a GC content below 50%. Based on the COA results, the sources of differences in synonymous codon usage among genes in Sapindaceae species are similar to those observed in Cucurbitaceae [20]. The first axis distinctly separated CDSs with a different GC content, indicating that mutations significantly influenced codon usage patterns in Sapindaceae species. COA analysis indicated that base composition contributed to shaping codon usage to some extent. Further investigation was needed into additional factors affecting codon usage bias, including gene expression length and RNA structure. These results suggested that mutation-driven base composition influenced the codon usage patterns among various Sapindaceae species, which is consistent with the findings in citrus [21] and Rosales species [22].

Mutation pressure and natural selection were recognized as the primary factors influencing codon usage bias (CUB) [23]. The ENC-GC3s plot analysis is commonly employed to assess these influences by examining the correlation between gene ENC values and GC3 content. In the eight Sapindaceae species, most genes were below the expected ENC plot curve, with only a few lying on or above it, suggesting that factors such as natural selection, rather than mutation, played a more dominant role. Additionally, analysis of the (ENCexp − ENCobs)/ENCexp values revealed that over 55% of CDSs exhibited ENC values deviating from the expected norms, indicating that mutation may play a minor role in the evolutionary history of Sapindaceae species, while natural selection likely had a more substantial influence on codon usage patterns. Similar results were also observed in *Ginkgo biloba* [24] and *Haloxylon ammodendron* [25]. To further determine the contribution of natural selection and mutational pressure to the CUB, a neutrality plot and PR2-Bias plot analysis were further used to elucidate this issue. Consistent with previous studies on other plants, a significant correlation between GC1/GC2 and GC3 was observed in the eight Sapindaceae species. Neutrality plot slopes ranged from 0.048 to 0.141 across the eight Sapindaceae species. Most slopes were similar to those observed in citrus (0.026–0.271) [21], Cucurbitaceae (0.074 to 0.153), and Rosale species (0.012–0.325) [22]. The neutrality plot analysis results indicated that natural selection predominates in the codon usage bias (CUB) of the eight Sapindaceae species. Furthermore, the PR2-Bias plot analysis showed that the average coordinates fell within the fourth quadrant, suggesting a notable preference for T- and G-ending codons at the third position of the CDSs of these species. Moreover, there was a possibility that GC3 had largely escaped the constraints of mutation pressure throughout the long process of natural selection [26]. The above results obtained from the neutrality, PR2-Bias, and ENC-GC3s plot analysis showed that natural selection was the primary driver of codon bias, while mutation played a lesser role.

Organisms utilize codons at different frequencies to encode the same amino acid [2]. Therefore, optimizing foreign gene sequences according to the target organism’s codon bias is essential for improving exogenous protein expression [27,28]. This optimization primarily seeks to reduce the use of rare codons, which increases transcription speed and decreases error rates [11]. This study identified 16 to 26 optimal codons per species, predominantly favoring A- and U-ending codons. This observation is consistent with the A/T-rich base composition found in many dicotyledons [9], suggesting that, under normal conditions, the optimal codons correlated with the GC and AT content of the genome [25]. In contrast to optimal codons that predominantly favored A/T endings, four NTA codons exhibited low RSCU values. It is well established that a reduction in TA may enhance protein production by mitigating mRNA degradation [29,30]. Furthermore, the low RSCU values of NCG codons may help prevent the potential mutations associated with DNA methylation [31,32]. Identifying optimal codons could offer valuable insights for gene editing aimed at improving in vivo expression across various Sapindaceae species.

## 4. Materials and Methods

### 4.1. Sequence Data Collection and Filtering

The coding sequences (CDSs) of eight typical Sapindaceae species, including *A. yangbiense* [33], *A*. *chinensis* [34], *C. halicacahum* [16], *S. mukorossi* [35], *X*. *sorbifolium* [36], *L. chinensis* [37], *N. lappaceum* [38], and *D. longan* [39], were retrieved from their respective genome databases. Detailed genome information about these eight Sapindaceae species can be found in Table S5. Subsequently, genome and annotation data for additional angiosperm species, including six monocots (*Brachypodium distachyon*, *Musa nana*, *Oryza sativa*, *Panicum virgatum*, *Sorghum bicolor*, *Zea mays*) and five dicot species (*Arabidopsis thaliana*, *P. trichocarpa*, *C. illinoinensis*, *C. Clementina*, *V. vinifera*) were downloaded from the Phytozome database [40].

CDSs shorter than 300 bp, lacking an ATG start codon, not ending with TAA, TAG, or TGA stop codons, containing uncertain nucleotides, or having internal stop codons were filtered using in-house Perl scripts (Table S6). Following filtering, high-quality sequences were utilized for subsequent analysis.

### 4.2. Codon Usage Bias and Related Indices Analysis

Each CDS’s overall GC content, along with the GC1, GC2, and GC3 values (indicating the GC content at the first, second, and third codon positions, respectively), reflected the balance between natural selection and mutation. RSCU (relative synonymous codon usage) measures the overall CUB among genes. An RSCU value of 1 indicates equal and random codon usage, while RSCU values greater than 1 and less than 1 signify a positive and negative codon usage bias (CUB), respectively [18]. ENC (effective number of codons) is used to assess the CUB of a gene, with values ranging from 20 to 61. An ENC value of 20 indicates that the amino acid is encoded by a single codon, reflecting the highest CUB, while an ENC value of 61 suggests no preference for synonymous codons [41]. The codon adaptation index (CAI) was employed to measure the similarity between a gene’s synonymous codon usage and the codon frequency of a reference set. CAI values range from 0 to 1, with a value of 1 indicating that a gene exclusively uses the most frequently used synonymous codons in the reference set [42]. In addition to the parameters mentioned above, we calculated the frequency of optimal codons (FOP), the number of synonymous codons (L_sym), the length of amino acids (L_aa), and the codon bias index (CBI) using the CodonW V l.4.2 (https://sourceforge.net/projects/codonw/, accessed on 1 May 2023) and CUSP (https://www.bioinformatics.nl/cgi-bin/emboss/cusp, accessed on 1 May 2023) programs.

### 4.3. ENC-GC3 Plot, PR2-Bias Plot, and Neutrality Plot Analysis

The ENC-GC3 plot was utilized to determine whether codon usage in specific genes was affected only by mutation or also by other factors, such as natural selection. The expected ENC values were plotted against GC3s values, calculated using the following formula [41]:

$$\mathrm{ENC}=2+\mathrm{GC}3s\;+\;\frac{29}{{\mathrm{GC}3s}^{2}{+(1\;-\;\mathrm{GC}3s)}^{2}}$$

In the ENC-GC3s plot, the x-axis represented GC3s values, while the y-axis displayed ENC values. If mutation pressure were the dominant factor influencing codon usage bias (CUB), then the data point would cluster closely around the expected curve. In contrast, if additional factors, such as selection, played a significant role in shaping CUB, the data points would be noticeably below or diverge from the expected curve.

The PR2 plot was generated using GC bias (G3/(G3 + C3)) and AT bias (A3/(A3 + T3)) values to evaluate the impacts of mutation and selection pressures. A value of 0.5 at the center of the plot indicated the absence of bias from selection or mutation between the two complementary DNA strands. Conversely, codon usage may be influenced by natural selection and other factors [43].

A neutrality plot, which displays GC12 against GC3, illustrates the balance between mutation and selection in shaping codon usage bias (CUB) [44]. GC12 represented the ratio of GC to AT content at the first and second codon positions. In these plots, if mutation pressure was the primary influence, a statistically significant correlation existed between GC12 and GC3, with the regression line’s slope approaching 1. Conversely, if natural selection was predominant, the GC content exhibited a narrow distribution, lacking a significant correlation between GC12 and GC3.

### 4.4. Correspondence Analysis

Correspondence analysis (COA), a multivariate statistical method employed in codon usage bias (CUB) analysis, was applied to RSCU values to investigate primary trends in codon usage variation among coding sequences (CDSs) and to map the codons onto axes representing these trends [45]. The COA was conducted using the RSCU values of codons with CodonW v 1.4.2 software.

### 4.5. Determination of Optimal Codons

ENC values of CDSs from each species were organized, separating the lowest and highest 10% to create high- and low-expression libraries. CDSs with low and high ENC values were clarified as high-expression and low-expression, respectively. RSCU and ΔRSCU values for each group were calculated. Codons with RSCU values greater than 1 were classified as high-frequency, while those with ΔRSCU values above 0.08 were classified as high-expression [18]. Codons satisfying both criteria were identified as optimal codons.

### 4.6. Codon Usage Analysis of the WRKY Gene Family in Sapindaceae Species

WRKY transcription factors have been identified as playing important roles in both primary and secondary metabolite biosynthesis [46]. Given the valuable nutritional and pharmaceutical properties of the eight selected Sapindaceae species, it is likely that WRKY transcription factors influence the biosynthesis of these valuable metabolites. To identify WRKY gene family members, we downloaded the HMM file for the WRKY protein domain (Pfam accession number PF03106) from the InterPro database [47] and used it to screen for potential candidates, with an E-value threshold of 10 × 10^−5^. The InterPro database was further used to confirm the presence of the WRKY domain. For the sequences predicted to belong to the WRKY gene family (Supplementary Material Table S3), we analyzed their codon usage bias using the method described above.

### 4.7. Comparison and Cluster Analysis

After excluding the 3 stop codons, the RSCU values for 59 codons from 8 Sapindaceae species and 11 other plants were clustered using Tbtools-II software (https://github.com/CJ-Chen/TBtools-II/releases, accessed on 1 May 2023) [48]. Hierarchical clustering and Euclidean distance were employed for the clustering process. Additionally, GC and GC3 variation values for the 19 species were analyzed.

## 5. Conclusions

The codon usage patterns of eight Sapindaceae species were systematically examined using their nuclear genomes. Nucleotide composition analysis confirmed that the genomes were AT-rich, favoring codons ending in T and/or A. Both natural selection and mutation shaped the CUB in the Sapindaceae species, with the former playing a more important role. Sixteen to 26 optimal codons were identified in the nuclear genomes, with AGA (Arg) and GCU (Ala) being the most commonly used across these species. Studying plant CUB and its influencing factors will enhance our understanding of the evolutionary history of these Sapindaceae species. Additionally, it will help identify the preferred optimal codons for applications in gene transfer and gene editing.

## Figures and Tables

**Table 1:** GC content and CDS across eight Sapindaceae species.

Species	GC	GC1	GC2	GC3	GC3s	The Number of CDSs
*Acer yangbiense*	43.62%	49.44%	39.64%	41.78%	39.46%	26,940
*Aesculus chinensis*	45.53%	50.57%	40.85%	45.17%	43.05%	28,312
*Cardiospermum halicacabum*	43.09%	49.71%	39.97%	39.59%	37.25%	33,483
*Dimocarpus longan*	44.32%	49.92%	40.38%	42.65%	40.38%	32,402
*Litchi chinensis*	43.84%	50.01%	40.18%	41.33%	39.07%	54,564
*Nephelium lappaceum*	44.56%	50.18%	40.46%	43.05%	40.84%	20,742
*Sapindus mukorossi*	43.75%	49.72%	41.06%	40.46%	38.13%	26,464
*Xanthoceras sorbifolium*	44.61%	50.28%	40.44%	43.13%	40.97%	23,500

**Table 2:** Frequency distribution of (ENCexp-ENCobs)/ENCexp. ENCexp represents expected ENC values and ENCobs represents ENC observed values.

Species	0.3~0.4	0.2~0.3	0.1~0.2	0~0.1	−0.1~0	−0.2~0.1
*Acer yangbiense*	0.41	12.97	28.14	60.21	7.44	0.15
*Aesculus chinensis*	0.43	14.08	24.73	61.71	9.44	0.20
*Cardiospermum halicacabum*	0.69	14.34	30.54	57.88	6.30	0.18
*Litchi chinensis*	0.40	12.41	27.98	59.87	8.07	0.19
*Dimocarpus longan*	1.34	19.19	28.40	55.41	9.09	0.30
*Sapindus mukorossi*	1.06	14.52	23.83	56.16	14.20	1.22
*Nephelium lappaceum*	0.47	13.22	29.29	58.42	7.77	0.20
*Xanthoceras sorbifolium*	0.39	12.18	26.14	62.49	7.63	0.16

**Table 3:** The top five high-frequency codons of 8 Sapindaceae species.

Species	Codon (RSCU)				
*Acer yangbiense*	AGA (2.087)	UUG (1.622)	GCU (1.619)	GUU (1.556)	UCU (1.516)
*Aesculus chinensis*	AGA (1.94)	AGG (1.534)	GCU (1.525)	UUG (1.519)	UCU (1.427)
*Cardiospermum halicacabum*	AGA (2.200)	GCU (1.782)	UCU (1.624)	CCU (1.624)	GUU (1.603)
*Litchi chinensis*	AGA (1.989)	GCU (1.647)	UUG (1.612)	GUU (1.570)	UCU (1.553)
*Dimocarpus longan*	AGA (2.001)	UUG (1.581)	GCU (1.572)	AGG (1.521)	GUU (1.494)
*Sapindus mukorossi*	AGA (1.837)	GCU (1.620)	UCU (1.526)	GUU (1.505)	UUG (1.503)
*Nephelium lappaceum*	AGA (1.990)	UUG (1.600)	GCU (1.589)	AGG (1.540)	GUU (1.511)
*Xanthoceras sorbifolium*	AGA (2.002)	GCU (1.567)	UUG (1.565)	GUU (1.533)	AGG (1.520)

## Data Availability

All data are contained within the article and its Supplementary Files.

## Supplementary Materials

The supporting information can be downloaded at: https://www.mdpi.com/article/10.3390/ijms26010039/s1.
