# Supplementary material for: Compare Analysis of Codon Usage Bias of Nuclear Genome in Eight Sapindaceae Species
Source: Int J Mol Sci. 2024 Dec 24;26(1):39. doi: 10.3390/ijms26010039 (PMC11720230; doi:10.3390/ijms26010039)
Supplement: Supplementary file 1 [file ijms-26-00039-s001.zip › Figures and Tables caption.pdf]

Figure S1: The Euclidean distance of the GC3 gradient between each pair of eight Sapindaceae species is calculated. A lower Euclidean distance indicates a closer relationship. The maximum and minimum values are highlighted in orange and red, respectively.
Figure S2: The GC and GC3 content across twenty-one species, including six monocots and thirteen dicot species, are analyzed. Purple rhombuses and green circles represent GC and GC3 content, respectively.
Table S1: Different indices used to evaluate the CUB of each CDS from the eight different Sapindaceae species.
Table S2: Optimal codons in nuclear genomes of eight Sapindaceae species.
Table S3: The WRKY gene family members of the eight different Sapindaceae species.
Table S4: High-frequency codons in the CDSs of WRKY gene family members across the eight Sapindaceae species.
Table S5: Detailed genome information about the eight Sapindaceae species.
Table S6: The Perl script was used to filter the appropriate CDSs in the eight species for further analysis.
