# Supplementary material for: Compare Analysis of Codon Usage Bias of Nuclear Genome in Eight Sapindaceae Species
Source: Int J Mol Sci. 2024 Dec 24;26(1):39. doi: 10.3390/ijms26010039 (PMC11720230; doi:10.3390/ijms26010039)
Supplement: Supplementary file 1 [file ijms-26-00039-s001.zip › Table S2.pdf]

Supplementary Table S2. Optimal codons in eight Sapindaceae species

Species	*Acer yangbiense*	*Aesculus chinensis*	*Cardiospermum halicacabum*	*Dimocarpus longan*	*Litchi chinensis*	*Nephelium lappaceum*	*Sapindus mukorossi*	*Xanthoceras sorbifolium*
Optimal codon numbers	25	16	26	19	23	18	25	20
Optimal codon	AGA	AGA	AGA	AGA	AGA	AGA	UUA	AGA
	UUG	AGG	UCU	UUG	UUG	UUG	UAA	AGG
	AGG	UCU	GCU	UCU	AGG	AGG	AGA	UCU
	GCU	UUG	UUG	AGG	GCU	GCU	UAU	GCU
	ACU	GCU	GUU	GCU	ACU	CCU	CGU	ACU
	CCU	ACU	CCU	GUU	GUU	UCU	ACU	UUG
	UCU	CCU	AUU	CCA	UCU	ACU	AGU	GUU
	GUU	GUU	CCA	CCU	CCU	GUU	AUU	CCU
	AUU	UCA	GGU	ACU	CAU	AGU	GCU	CCA
	CCA	AUU	ACU	AUU	CCA	AUU	CAA	AUU
	CAU	CCA	UGU	AGU	AUU	CAU	GUU	CAU
	AGU	GGU	CAU	GGU	GAU	GAU	GAU	GAU
	GAU	CAU	AAU	CAU	AGU	CCA	UUU	GGU
	GGU	GAU	ACA	UCA	UCA	UCA	AAA	UCA
	UUU	UGA	GAU	GAU	AAU	ACA	GUA	CUU
	UGU	AAU	AGU	GCA	UUU	UUU	GAA	UUU
	ACA		AGG	UGU	UAU	UGU	GGA	AAU
	UCA		UCA	GGA	UGU	GGU	AAU	GCA
	UAU		UAU	UAU	GGU		UCU	UGU

GCA	UUU	ACA	CUU	AGU
AAU	UUA	GCA	CGA	
UUA	CAA	GGA	CCA	
CAA	GCA	UAG	CAU	
GGA	GGA		UUG	
CUU	CUU		GCA	
	GAA			
