# Supplementary material for: Compare Analysis of Codon Usage Bias of Nuclear Genome in Eight Sapindaceae Species
Source: Int J Mol Sci. 2024 Dec 24;26(1):39. doi: 10.3390/ijms26010039 (PMC11720230; doi:10.3390/ijms26010039)
Supplement: Supplementary file 1 [file ijms-26-00039-s001.zip › Table S6.pdf]

```
use Bio::SeqIO;

# Set the input file path
my $input_file = $ARGV[0];

# Check if the input file exists
open my $fh, $input_file or die "Cannot open file: $input_file";

# Create a sequence input object
my $seq_io = Bio::SeqIO->new(-file => $input_file, -format => 'fasta');

while (my $seq_obj = $seq_io->next_seq()) {
    my $id = $seq_obj->id;
    my $sequence = $seq_obj->seq;
    my $description = $seq_obj->desc;
    my @description_parts = split("=|]", $description);
    my $length = length($sequence);
    my $start_codon = substr($sequence, 0, 3);
    my $end_codon = substr($sequence, -3);
    if ($start_codon eq "ATG" && $length > 300 &&
        ($end_codon eq "TAA" || $end_codon eq "TAG" || $end_codon eq "TGA")) {
        print ">$description_parts[1]\t$length\n$sequence\n";
    }
}
```
