# Supplementary figures and images for: Compare Analysis of Codon Usage Bias of Nuclear Genome in Eight Sapindaceae Species
Source: Int J Mol Sci. 2024 Dec 24;26(1):39. doi: 10.3390/ijms26010039 (PMC11720230; doi:10.3390/ijms26010039)

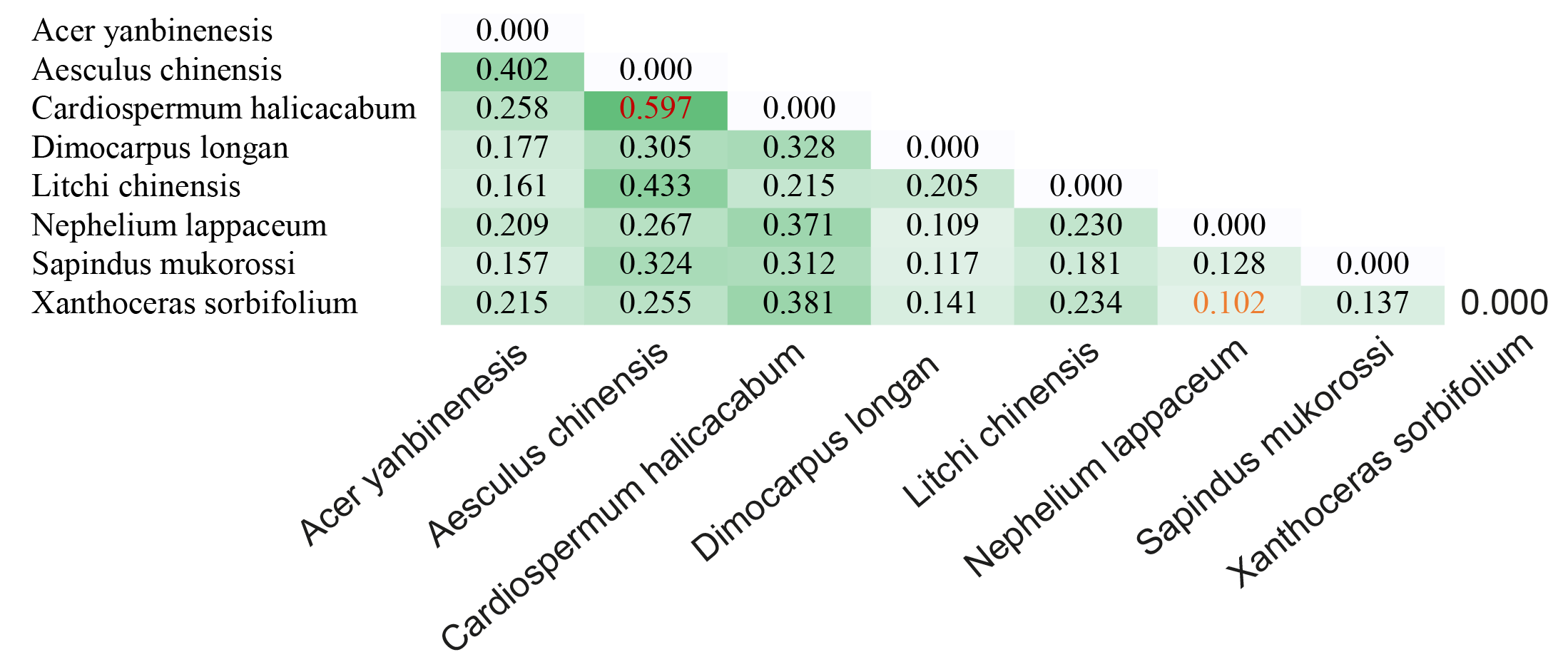

Supplement: Supplementary file 1 [file ijms-26-00039-s001.zip › Figure S1.tif]

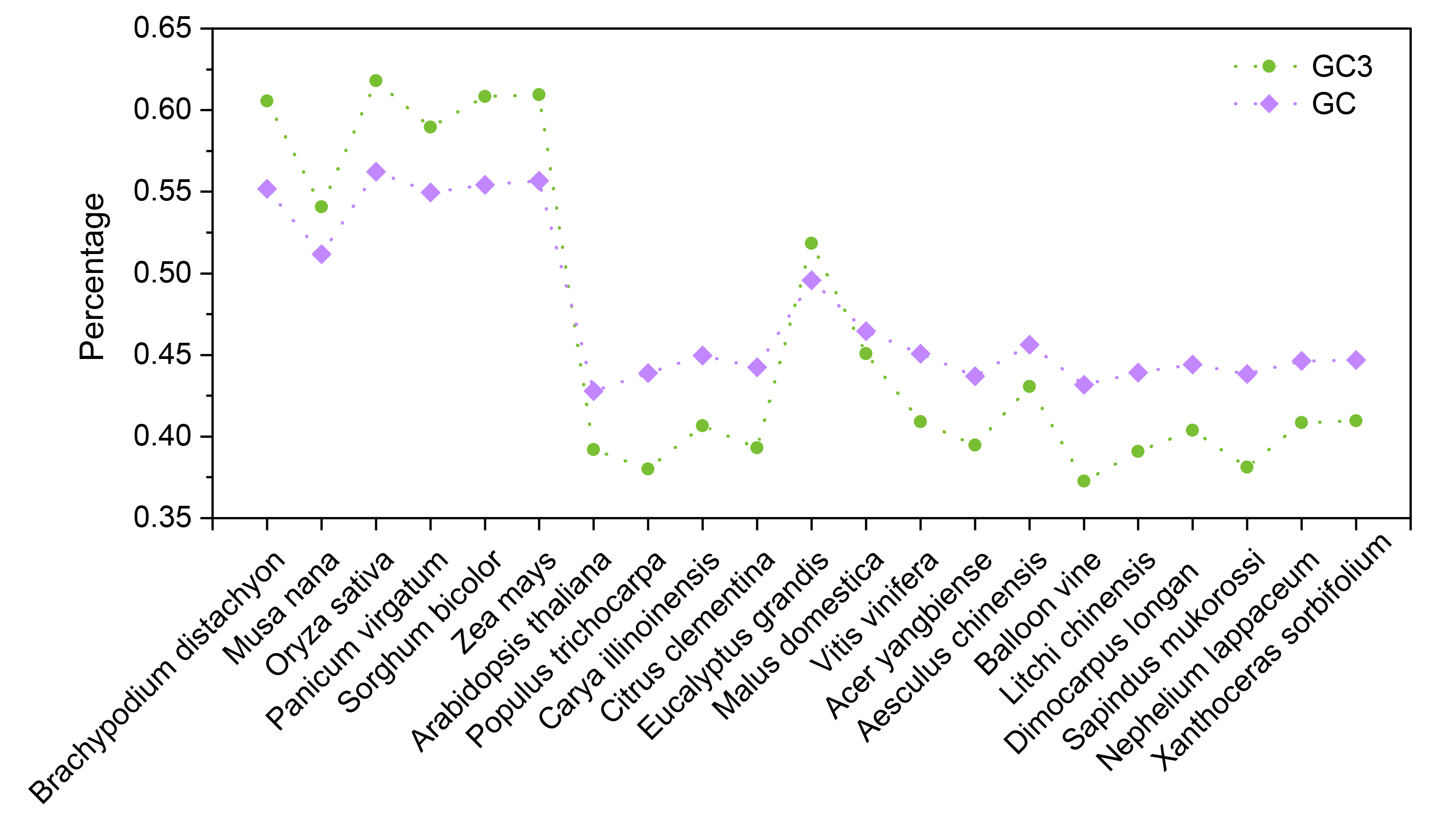

Supplement: Supplementary file 1 [file ijms-26-00039-s001.zip › Figure S2.tif]
